# The validity and reliability of quality of life questionnaires in patients with ankylosing spondylitis and non-radiographic axial spondyloarthritis: a systematic review and meta-analysis

**DOI:** 10.1186/s12955-022-02026-5

**Published:** 2022-07-30

**Authors:** Qian He, Jing Luo, Jiaqi Chen, Jianying Yang, Chuanhui Yao, Caiqin Xu, Qingwen Tao

**Affiliations:** 1 Graduate School, Beijing University of Chinese Medicine, Beijing, 100029 China; 2 Traditional Chinese Medicine Department of Rheumatism, China-Japan Friendship Hospital, Beijing, 100029 China; 3 Beijing Key Lab for Immune-Mediated Inflammatory Diseases, China-Japan Friendship Hospital, Beijing, 100029 China

**Keywords:** Ankylosing spondylitis, Non-radiographic axial spondyloarthritis, Questionnaire, Systematic review, Meta-analysis

## Abstract

**Background:**

Patients who suffered from ankylosing spondylitis (AS) or non-radiographic axial spondyloarthritis (nr-axSpA) often have poor quality of life (QoL) and there has been a substantial increase in research on acceptable questionnaires for assessment of QoL. This systematic review aims at examining the validity and reliability of QoL questionnaires in patients with AS/nr-axSpA.

**Methods:**

Randomized controlled trials (RCTs), cohort trials, and cross-sectional trails were retrieved by searching seven databases. Primary outcomes included test–retest reliability and construct validity. Secondary outcomes included internal consistency, structural validity, responsiveness and so on. Data extraction and analyses were conducted according to the Cochrane standards. The Agency for Healthcare Research and Quality (AHRQ) checklists was used to assess the risk of bias for each included study. We used the Consensus-based Standards for the Selection of Health Status Measurement Instruments (COSMIN) to assess the methodological quality and measurement property of included instruments. The quality of evidence on pre-specified outcomes were assessed by the Grades of Recommendations, Development and Evaluation (GRADE) approach.

**Results:**

22 publications containing 10 self-rating instruments were included in this study. Most studies were cross-sectional in design and a total of 3,085 participants were enrolled. 19 studies had moderate to high test–retest reliability. Cronbach’s alpha (α) Coefficients were generally high (0.79–0.97) for overall scales. The ankylosing spondylitis quality of life (ASQOL) and evaluation of ankylosing spondylitis quality of life (EASi-QoL) questionnaires showed the strongest measurement properties in high-quality studies. The correlation coefficient for test–retest reliability of the ASQOL questionnaire was 0.85 (95% CI 0.80 to 0.89). The pooled Cronbach’s α coefficients of the ASQOL questionnaire and the EASi-QoL questionnaire were high. Bath Ankylosing Spondylitis Disease Activity Index (BASDAI) and Bath Ankylosing Spondylitis Functional Index (BASFI) were considered as two validity criteria. For the ASQOL and EASi-QoL questionnaire, pooled convergent validity associations with BASDAI and BASFI were low to strong (0.24–0.81).

**Conclusions:**

This study indicated acceptable reliability and stability of included QoL questionnaires. The ASQOL and the EASi-QoL questionnaires are validated and reliable disease-specific questionnaires for the assessment of QoL in patients with AS/nr-axSpA.

**Supplementary Information:**

The online version contains supplementary material available at 10.1186/s12955-022-02026-5.

## Introduction

Ankylosing spondylitis (AS) and non-radiographic axial spondyloarthritis (nr-axSpA) are common chronic inflammatory arthritis affecting the axial skeleton [1], which is characterized by chronic low back pain, radiographic sacroiliitis, excess spinal bone destruction and aberrant bone formation, and generally with positive HLA-B27. Current estimates indicate that AS affects up to 0.1–1.4% of the adult population worldwide [2], while data for nr-axSpA is not currently available. An update of review shows that the prevalence of AS ranged from 9 to 30 per 10,000 persons, and the risk of mortality seems to be increased [3]. The prevalence of AS was higher in males compared with females [4], with gender ratios of around 3.8:1 in Europe and 2.3:1 in Asia [2].

According to 2019 ACR recommendations, the primary recommendations of medical treatment is nonsteroidal anti-inflammatory Drugs (NSAIDs) and tumor necrosis factor inhibitors (TNFi) for AS/nr-SpA [5]. Moreover, non-pharmacological managements such as back exercise also have a benefit for releasing back pain and morning stiffness. However, there remains approximately 40% of patients do not achieve adequate disease control [6]. Bone destruction and aberrant often result in serious impairment of spinal mobility and physical function in patients with AS/nr-SpA. The onset is usually in early adulthood, even in late adolescence. Patients with AS/nr-SpA often have to suffer from disability during the most of life, and incapacity for work. Meanwhile, social problems, depression, and sexual activity difficulty have been reported among patients with AS/nr-SpA. Thus, sufficient shreds of evidence remind that patients with AS/nr-SpA often have poor health-related quality of life (QoL).

The world health organization (WHO) definition of QoL contains physical, psychological, and social [7]. As the increasing concerns of QoL, it has become an important outcome in studies. The measurements of QoL may make a contribution to improve health care, evaluate the safety of some specific therapies, predict disease activity and elucidate proper targets for treatment. Several tools have been developed to assess the patients’ self-reported QoL, which include the generic Short Form-36 (SF-36) survey, the world health organization quality of life (WHOQoL) pilot instrument, the disease-specific ankylosing spondylitis quality of life (ASQOL) [8] questionnaire and the evaluation of ankylosing spondylitis quality of life (EASi-QoL) [9] questionnaire. There is a glaring absence of a systematic review or meta-analysis concentrating on the comparative reliability and validity of QoL questionnaires in recent five years. The aim of this systematic review is to fill the information gap.

## Methods and analysis

This systematic review is reported according to the Preferred Reporting Items for Systematic Reviews and Meta-Analyses (PRISMA) guidelines checklist.

### Protocol and registration

The protocol of this systematic review is documented in PROSPERO (ID = CRD42021218489).

### Eligibility criteria

#### Types of study

Studies will be included if they use questionnaires assessing the QoL for patients with AS/nr-SpA, with no restrictions of language, and years of publishment. Randomized controlled trials (RCTs), cohort trials, and cross-sectional trails will be included.

#### Types of participants

Adult patients (≥ 18 years old) meet the standardized diagnostic criteria, such as the assessment of spondyloarthritis international society (ASAS) imaging criteria for axSpA [1] or the 1984 modified New York criteria for AS/nr-SpA [8], with no restrictions of gender or ethnicity.

#### Types of outcome measure

##### Primary outcomes

The test–retest reliability, and construct validity of the included health-related QoL questionnaires.

##### Secondary outcomes

The internal consistency, structural validity, responsiveness, and the floor and ceiling effects of included QoL tools.

### Exclusive criteria

1. Conference abstract, editorial, opinion article, scientific statement, guideline, protocol, animal trials, retraction, review, or duplicate publications.
2. Studies could not provide available data.

### Search methods

#### Electronic searches

The following online databases were searched from inception to October 31, 2020: PubMed, EMBASE, the Cochrane Library, China National Knowledge Infrastructure (CNKI), Chinese Scientific Journal Database (VIP), Wanfang Database, and SinoMed Database. The following search terms were used: ankylosing spondylitis, axial spondyloarthritis, quality of life, reliability, validity, internal consistency, questionnaires, surveys, scales, index, SF-36, and short forms, both in Chinese and English.

#### Searching other resources

We also screened reference lists of retrieved articles to identify potential missing studies.

### Search strategies

Details of search strategies in English databases were provided in Additional file 1.

### Study selection

The title/abstract and full article were screened by two reviewers (JQ Chen, JY Yang) according to the eligible criteria independently. Disagreements were resolved by consensus, or discussion with the third review author (J Luo). The full selection process was presented in a flow diagram.

### Data extraction

A predesigned data extraction form was used to extract relevant data by four reviewers (JQ Chen, JY Yang, CH Yao, and CQ Xu) independently. The following information was included:

1. General information (title, the first author, year of publication, funding, country, study design, sample size, setting)
2. Participants (disease, diagnostic standard, age, gender, disease duration)
3. Properties of target questionnaires (instruments and version, number of items, internal consistency, test–retest reliability, convergent validity and discriminative validity, structural validity)

The missing information was sought by contacting the original authors if possible. Any discrepancies were resolved by consulting a third reviewer (J Luo).

### Quality assessment

#### Risk of bias assessment

The tool recommended by the Agency for Healthcare Research and Quality (AHRQ) [10] was adopted to assess the risk of bias of include studies. The following criteria were assessed: selection bias and confounding, performance bias, attrition bias, detection bias, reporting bias, and other bias (risk of bias graph was provided in Additional file 2). Each item was judged as low risk of bias, high risk of bias or unclear on consensus between two reviewers (Q He and JQ Chen). Disagreement was resolved by consulting a third reviewer (J Luo).

#### Evaluation of the methodological quality and measurement property

Firstly, the risk of bias checklist of the uniform criteria tools (Consensus-based Standards for the selection of health status Measurement Instruments, COSMIN [11]) was used to assess the methodological quality of instruments. Each item was rated as very good, adequate, doubtful and inadequate. Then, two separate authors (Q He and JQ Chen) awarded a score of either positive (+), negative (−) or indeterminate (?) to each measurement property, based upon the quality criteria for good measurement criteria. At last, the reviewers graded the quality of evidence by the Grading of Recommendations Assessment, Development, and Evaluation (GRADE) approach [12]. The quality of evidence for each outcome was judged as high, medium, low, extremely low. Disagreement was discussed with a third reviewer (J Luo). Details is given in Additional file 2.

### Statistical analysis and data synthesis

Meta-analysis of extracted coefficients of reliability and validity was performed when data was available from at least two studies. The Chi-square test was conducted to test heterogeneity. If heterogeneity was noticeable (50% < I^2^ < 75%), a random-effects model was performed to pool the effect sizes. If the I^2^ value was low (I^2^ ≤ 50%), a fixed-effects model will be performed. Data were not pooled when heterogeneity was high (I^2^ ≥ 75%). Subgroup analysis was adopted to explore potential reasons for heterogeneity according different characteristics. Sensitivity analysis was conducted if the heterogeneity was significant. Funnel plots were used to detected the publication bias if studies were more than eight.

Quantitative synthesis was conducted with Stata V.16.0 software. Internal consistency was reported as Conbranch's alpha (α) coefficient and test–retest reliability was reported as intraclass correlation coefficient (ICC) or Spearman’s correlation coefficients. All coefficients were transformed when meta-analysis was conducted. Conbranch's α coefficient and ICC were transformed with the method proposed by Hakstian and Whalen [13] ($Transformed\;Conbranch^{\prime}s\;\alpha = \mathop {\left( {1 - \alpha } \right)}\nolimits^{\frac{1}{3}}$). Spearman’s or Pearson’s correlation coefficients was transformed to Fisher's Z scales (①$\mathop r\nolimits_{s} = \frac{6}{\Pi }\mathop {\sin }\nolimits^{ - 1} \left( \frac{2}{r} \right)$ (r_s_ = Spearman’s correlation coefficients, r = Pearson’s correlation coefficients); ②$fisher^{\prime}s\;Z = 0.5 \times In\left( {\frac{{1 + \mathop r\nolimits_{s} }}{{1 - \mathop r\nolimits_{s} }}} \right)$; ③ $\mathop v\nolimits_{z} = \frac{1}{N - 3}$; ④ $\mathop s\nolimits_{E} = \sqrt {\mathop v\nolimits_{z} }$). The pooled effects and confidence interval were transformed back to the original scale ($Summary\;r = \frac{{\mathop e\nolimits^{2z} { - }1}}{{\mathop e\nolimits^{2z} + 1}}\left( {Z = Summary\;fisher^{\prime}s\;Z} \right)$) to evaluate the measurement properties of QoL tools.

A ‘Summary of findings’ table was created using the GRADE profiler (V.3.6.1). Detailed description of correlation coefficients and Fisher’s Z calculations is given in Additional file 2. Funnel plots are given in Additional file 3.

## Results

### Study selection

**Fig. 1:**
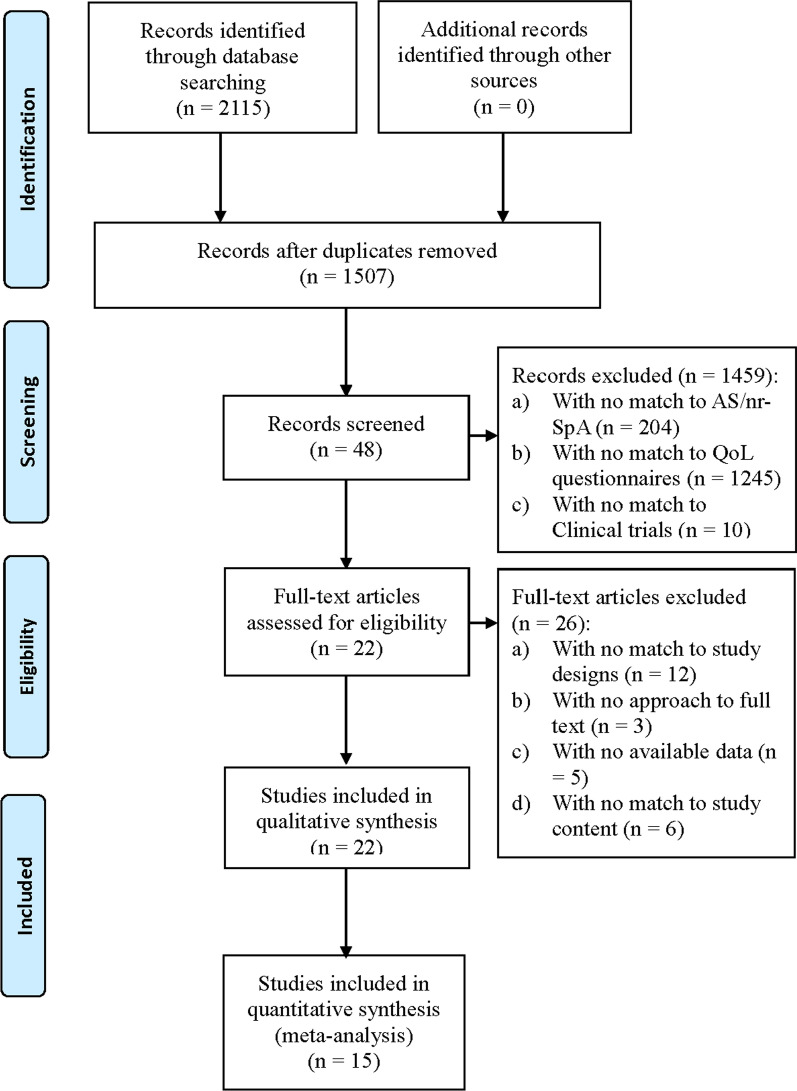
Flow diagram of study search and identification

2115 publications were retried in the search, including 608 duplicate publications which were removed. 1459 articles were excluded, 1449 of them didn’t focus on our topic, and 10 articles were not clinical trials. As a result, 48 publications were selected after the title and abstract screening. 22 articles [8, 14–34] were enrolled according to the inclusion criteria and 15 [8, 15–25, 28, 29, 31] of them were included in meta-analysis. A PRISMA flow diagram was created to describe the study selection process (Fig. 1).

### Characteristics of included studies

Characteristics of studies included in the literature review were provided in Table 1. Two articles [32, 34] were published in Chinese, with one in Spanish [19] and one in French [24], and the others were English publications. 18 studies [8, 14, 17–22, 24–32] were cross-sectional in design, two [33, 34] were cohort studies and two [16, 23] were RCTs. Data from 3,085 participants was extracted at an average disease duration of 15 years. The percentage of male was ranging from 28.9 to 89.0%.

**Table 1:** Characteristics of studies included in this systematic review

Reference	Study design	Country (language)	HRQoL tools	Patients’ characteristics				PROM properties			
				Sample size(N)	Age (Y, M ± SD)	Male N (%)	Disease duration (Y, M ± SD)	Internal consistency	COSMIN (MQ)	Test–retest reliability	COSMIN (MQ)
Doward et al. [8]	cross-sectional study	UK(English)	ASQOL	129	(46.10 ± 12.40)	93 (72%)	19.60	0.91;0.92	Inadequate	r = 0.92	Adequate
		Netherlands (English)	ASQOL	119			20.80	0.89;0.90		r = 0.91	
Haywood et al. [14]	cross-sectional study	England (English)	EuroQol	451(349)	(46.10 ± 12.60)	259 (74.2%)	NR	NR	NR	closed format: ICC = 0.88 blind format: ICC = 0.82	Adequate
	cross-sectional study	England (English)	SF-12	451 (349)	(46.10 ± 12.60)	259 (74.2%)	NR	0.91	Very good	NR	NR
Jenks et al. [15]	cohort study	New Zealand (English)	ASQOL	63	43.30	40 (63%)	8.90	0.854	Very good	rho = 0.73	Very good
Doward et al. [16]	RCT	United States (English)	ASQOL	148	(44.70 ± 12.50)	111 (75%)	(11.00 ± 10.30)	0.85	Very good	r = 0.85	Very good
Duruöz et al. [17]	cross-sectional study	Turkish (English)	ASQOL	277	(42.20 ± 11.60)	80 (28.9%)	(9.40 ± 9.40)	0.89	Very good	ICC = 0.96	Adequate
Leung et al. [18]	cross-sectional study	Singapore (English)	ASQOL	183	(39.50 ± 13.70)	141 (77%)	NR	Chinese: α = 0.93 English: α = 0.86	Very good	ICC = 0.86	Very good
Ariza-Ariza et al. [19]	cross-sectional study	Spain (Spanish)	ASQOL	54	(40.50 ± 10.50)	37 (68.5%)	NR	0.86	Very good	r = 0.98	Very good
Haywood et al. [20]	cross-sectional study	England (English)	ASQOL	271	(46.10 ± 12.60)	259 (74.2%)	NR	0.92	Very good	ICC = 0.96	Very good e
	cross-sectional study	England (English)	RLDQ	179	(46.10 ± 12.60)	259 (74.2%)	NR	0.93	Very good	ICC = 0.95	Adequate
Fallahi et al. [21]	cross-sectional study	Iran (English)	ASQOL	163	(37.74 ± 9.88)	129 (79%)	(14.49 ± 8.47)	0.91	Very good	ICC = 0.97	Adequate
Pham et al. [22]	cross-sectional study	French (English)	ASQOL	139	(40.90 ± 13.70)	76 (54.6%)	(13.10 ± 11.30)	0.9	Very good	ICC = 0.89	Adequate
Zhao et al. [23]	RCT	China (English)	ASQOL	18	(31.80 ± 8.80)	102 (89%)	(8.80 ± 7.00)	NR	NR	NR	NR
Hamdi et al. [24]	cross-sectional study	Tunisian (French)	ASQOL	18	(38.35 ± 12.26)	84 (84.8%)	(11.30 ± 9.40)	0.93 (0.86–0.95)	Very good	ICC = 0.875(0.79–0.92)	very good
Hoepken et al. [25]	cross-sectional study	Germany (English)	ASQOL	37	(41.90 ± 11.80)	26 (70.3%)	(9.70 ± 9.10)	0.79	Very good	r = 0.77	very good
Kwan et al. [26]	cross-sectional study	Singapore (English)	SF-36	22	(40.70 ± 10.80)	16 (72.7%)	(9.20 ± 9.40)	0.87	Very good	r = 0.84	very good
Haywood et al. [27]	multicenter cross-sectional survey	Spain (English)	PGI	24	(38.00 ± 9.00)	19 (79.2%)	(12.20 ± 8.90)	0.84	NR	r = 0.77	very good
Öncülokur et al. [28]	cross-sectional study	Sweden (English)	EASi-QoL	9	(37.60 ± 9.10)	8 (88.9%)	(11.40 ± 9.40)	0.81	Very good	r = 0.85	Very good
Haywood et al. [29]	cross-sectional study	England (English)	EASi-QoL	612	(50.80 ± 12.20)	434 (71.6%)	(17.30 ± 11.70)	PF:0.90 DA:0.88 EW:0.91 SP:0.92	Very good	PF: ICC = 0.93 DA: ICC = 0.88 EW: ICC = 0.90 SP: ICC = 0.90	Adequate
El Miedany al. [30]	cross-sectional study	Arabic (English)	CASQ-QoL	122	(38.90 ± 8.70)	NR	(12.10 ± 4.20)	0.96–0.97	Very good	r = 0.9	Very good
Graham et al. [31]	cross-sectional study	Greece (English)	ASQOL	92 (87)	(49.60 ± 11.50)	63 (68.5%)	NR	0.92	Very good	r = 0.98	Doubtful
Liu et al. [32]	cross-sectional study	China (Chinese)	SQOL-AS	37	(28.12 ± 7.63)	50 (84.7%)	NR	0.54–0.91	Very good	NR	NR
Boonen et al. [33]	cohort study	Netherlands (English)	EQ-5D	254	(41.40 ± 13.70)	8 (80%)	(10.90 ± 5.70) vs (14.90 ± 9.30)	NR	NR	ICC = 0.55	Doubtful
			SF-6D							ICC = 0.68	
Guilleminet al. [34]	cohort study	China (Chinese)	modified AS-AIMS2	146	(47.30 ± 12.80)	98 (67.1%)	(18.10 ± 11.90)	0.78–0.91	Very good	Physical: ICC = 0.90, Affect: ICC = 0.70, Symptoms: ICC = 0.81, Role: ICC = 0.81, Social interaction: ICC = 0.90	Doubtful

HRQoL, health-related quality of life; ASQOL, the ankylosing spondylitis quality of life questionnaire; EASi-QoL, the evaluation of ankylosing spondylitis quality of life questionnaire; RLDQ, the revised Leeds disability questionnaire; CASQ-QOL, the combined AS questionnaire for quality of life questionnaire; PGI, the patient-generated index; SF-36, the short form-36 health survey; SF-12, the short form-12 health survey; modified AS-AIMS2, the ankylosing spondylitis-arthritis impact measurement scales 2; SQOL-AS, an ankylosing spondylitis patient quality of life measurement scale. PF, physician function; DA, disease activity; EW, emotional well-being; SP, social participation; ICC, intraclass correlation coefficient; RCTs, randomized controlled trials; NR, not report

A total of 12 self-rating instruments were included in this study. 13 studies [8, 15–25, 31] administered the ASQOL questionnaire, two [28, 29] assessed the EASi-QoL questionnaire, one reported the revised Leeds disability questionnaire (RLDQ) [20], the combined AS questionnaire for quality of life (CASQ-QoL) [30] questionnaire, the EuroQol [14] questionnaire, the patient-generated index (PGI) [27], the short form-36 health survey (SF-36) [26], the short form-12 health survey (SF-12) [14], and the modified ankylosing spondylitis-arthritis impact measurement scales 2 (AS-AIMS2) [34] respectively. One study attempted to develop an AS patient quality of life measurement scale (SQOL-AS) [32] in the Chinses population, and another one focused on comparing the characteristics of the EQ-5D and SF-6D scales [33].

All the questionnaires were convenient to finish within 10 min and the ASQOL questionnaire usually took out 2.4 to 5 min. The cross-cultural adoption of these instruments was confirmed. The disease-specific ASQOL questionnaire as well as the general SF-36 could multidimensionally assess the QoL in patients, including physical functioning, role physical, bodily pain, general health, vitality, social function, role emotion, and mental health. Physical component summary (PMC) and mental component summary (MCS) were set to summarize the physical and mental health. The SF-12 survey is a simplified version of the SF-36 survey, which maybe more suitable to report the QoL in general population or evaluate the change of condition in spectacular patients. The AS-AIMS2 and RLDQ had concentrated on disabling conditions, while the modified AS-AIMS2 exploring the aspects of mental health, emotional well-being and social interactivity. The PGI has been validated to estimate the life expectancy of patients and nominated areas of their life affected by disease. Properties of included instruments is given in Additional file 2.

### Risk of bias in the included studies

**Fig. 2:**
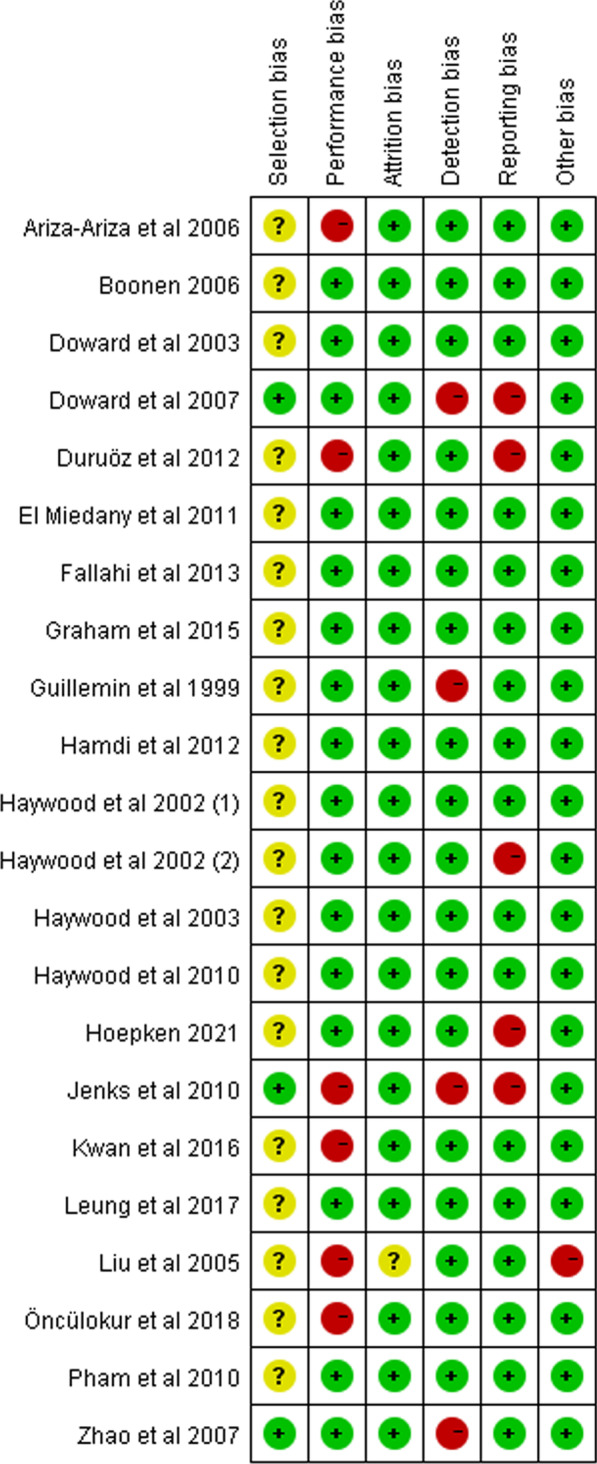
Risk of bias summary of include studies

Risk of bias summary for each study was shown in Fig. 2. The overall risk of bias was evaluated as low. Regarding the individual studies, it shows that performance bias and reporting bias are the majority of the risk of bias. Risk of bias graph is given in Additional file 3.

### Evaluation of the methodological quality and measurement property

According to the COSMIN criteria, 18 [14–22], [24–26], [28–32], [34] studies were found to have “very good” methodological quality of internal consistency and one [8] has “inadequate”. 11 studies [15, 16, 18–20, 24–28, 30] was rated as “very good”, 6 [8, 14, 17, 21, 22, 29] as “adequate” and 3 [31, 33, 34] as doubtful in the methodological quality evaluation of reliability. 11 [8, 14, 16, 18, 20, 22, 28–31] studies were found having “adequate” methodological quality for structural validity. Methodological quality evaluations concerning construct validity of 7 studies [8, 15, 16, 19, 20, 28, 29] was undertaken and received a “very good” methodological quality rating. The pooled results of per patient-reported outcome measures (PROMs) rated against the same quality criteria for good measurement properties were shown in Table 2 (Details are showed in Additional file 2).

**Table 2:** Methodological quality of PROMs and quality of measurement properties

PROM (ref)	Country /language in which the PROM was evaluated	Structural validity			Internal consistency			Reliability			Construct validity		
		n	Meth qual	Result (rating)	n	Meth qual	Result (rating)	n	Meth qual	Result (rating)	n	Meth qual	Result (rating)
ASQOL [8, 15–25, 31]	New Zealand, United States, Canada, Germany, France, Italy, Spain, Sweden, Turkish, Chinese, Singapore, Spain, Netherlands, Persian, Greek	1650	?	⨁⨁⨁⊝ Moderate	1929	+	⨁⨁⨁⨁High	1348	+	⨁⨁⨁⨁High	1830	?	⨁⨁⨁⊝ Moderate
EASi-QoL [28, 29]	Turkish, England	712	±	⨁⨁⨁⊝ Moderate	712	+	⨁⨁⨁⊝ Moderate	712	+	⨁⨁⨁⊝ Moderate	100	+	⨁⊝⊝⊝ Very low
EuroQoL [14]	England	321	−	⨁⊝⊝⊝ Very low	321	?	⨁⊝⊝⊝ Very low	176	+	⨁⊝⊝⊝ Very low	176	+	⨁⊝⊝⊝ Very low
SF-12 [14]	England	321	−	⨁⊝⊝⊝ Very low	321	?	⨁⊝⊝⊝ Very low	321	+	⨁⊝⊝⊝ Very low	321	?	⨁⊝⊝⊝ Very low
SF-36 [26]	England	196	−	⨁⊝⊝⊝ Very low	196	±	⨁⊝⊝⊝ Very low	NR	NR	NR	196	±	⨁⊝⊝⊝ Very low
PGI [27]	England and Scotland	343	?	⨁⊝⊝⊝ Very low	NR	?	⨁⊝⊝⊝ Very low	69	+	⨁⊝⊝⊝ Very low	343	?	⨁⊝⊝⊝ Very low
CASQ-QoL [30]	Arabic	122	?	⨁⊝⊝⊝ Very low	122	+	⨁⊝⊝⊝ Very low	122	+	⨁⊝⊝⊝ Very low	NR	NR	NR
RLQD [20]	England	271	?	⨁⊝⊝⊝ Very low	271	+	⨁⊝⊝⊝ Very low	179	+	⨁⊝⊝⊝ Very low	271	+	⨁⊝⊝⊝ Very low
SQOL-AS [32]	China	37	NR	NR	37	+	⨁⊝⊝⊝ Very low	NR	NR	NR	NR	NR	NR
EQ-5D	Netherlands	NR	NR	NR	NR	NR	NR	NR	NR	NR	NR	NR	NR
SF-6D	Netherlands	NR	NR	NR	NR	NR	NR	NR	NR	NR	NR	NR	NR
Modified AS-AIM2 [34]	Lorraine region	146	?	⨁⊝⊝⊝ Very low	146	+	⨁⊝⊝⊝ Very low	43	+	⨁⊝⊝⊝ Very low	NR	NR	NR

+, sufficient; −, insufficient; ?, indeterminate; ±, inconsistent; NR, not report

### Test–retest reliability

19 studies [8, 14–22, 24–31, 34] had moderate to high test–retest reliability (ICC value of 0.82 to 0.96 or Spearman’s correlation coefficients of 0.70 to 0.98). Adequate to very good test–retest reliability of high quality of evidence was found for the ASQOL questionnaire (ICC values of 0.44 to 0.933), and very good reliability of moderate quality was found for the EASi-QoL questionnaire (ICC range from 0.88 to 0.935) [28, 29], the RLDQ (ICC = 0.95) [20], the CASQ-QoL (r = 0.9), the Euro-QoL questionnaire (closed format: ICC = 0.88, blind format: ICC = 0.82) [14], the EQ-5D scale (ICC = 0.55) [33], the SF-6D scale (ICC = 0.68) [33], and the PGI(closed format: ICC = 0.88,blind format: ICC = 0.82) [27].

A sensitivity analysis was performed to reduce the significant heterogeneity (values of 88.6% to 60.47%). The pooled Fisher's Z estimate of the ASQOL scales was 1.26 (95% CI 1.10 to 1.41), and the pooled correlation coefficient value of test–retest reliability was 0.85 (95% CI 0.80 to 0.89).

### Construct validity

Construct validity indicating the associations with the validity criteria includes convergent validity and discriminative validity. The Bath Ankylosing Spondylitis Disease Activity Index (BASDAI) and Bath Ankylosing Spondylitis Functional Index (BASFI) are commonly used to assess the disease activity of patients with AS/nr-SpA. The convergent validity of the ASQOL questionnaire is weak to good. The summary r values of the association with ASQOL questionnaire and BASDAI were 0.78 (95% CI 0.74 to 0.82) and 0.54 (95% CI 0.47 to 0.61) in the Europe and regions beyond Europe. Subgroup analysis demonstrated that the ASQOL questionnaire was more validated and reliable to evaluate the QoL in the Europe than other regions. The pooled summary r value of association with ASQOL questionnaire and BASFI was 0.62 (95% CI 0.57 to 0.68). The funnel plot had symmetry. The EASi-QOL questionnaire focuses on four dimensions: physician function, disease activity, emotional well-being, and social participation. “Very good” to convergent validity of moderate evidence quality of the association with scores of BASFI and the ASQOL questionnaire were 0.65 (95% CI 0.71 to 0.75) and 0.70 (95% CI 0.64 to 0.74).

### Internal consistency

In 20 studies [8, 14–22, 24–32, 34], internal consistency of most included scales was generally high, with Cronbach’s α coefficients values of 0.79 to 0.97. Only one [16] study reported poor internal consistency with a Cronbach’s α coefficient value of 0.44. The ωH value of 0.82 was reported as a measure of reliability in one article [25].

The overall effects of transformed Cronbach’s α coefficient of the ASQOL and EASi-QoL questionnaires were 0.48 (95% CI 0.43 to 0.52), 0.46 (95% CI 0.42 to 0.49). The pooled Cronbach’s α coefficients scored as high and moderate quality of evidence of these two scales were 0.89 (95% CI 0.86 to 0.92), 0.91 (95% CI 0.88 to 0.93). It indicates good internal consistency and stability. No heterogeneity (I^2^ = 0.0) was detected, so the fix-effects model was chosen to conduct the meta-analysis.

### Structural validity

Item response theory (IRT)/Rasch model has been used in five articles to test the structural validity of the ASQOL and EASi-QoL questionnaires. Two publications [28, 29] chose the exploratory factor analysis (EFA) and confirmatory factor analysis (CFA) models for the EASi-QOL questionnaire. It showed that the factor loadings were higher than 0.40 and the item-total correlations were ranged from 0.66 to 0.84. Principle component analysis (PCA) [20] was performed in five studies to assess the dimensionality of instruments. A 2-parameter Rasch model confirmed unidimensionality (chi-square fit *p* = 0.86) with good item discrimination of the RLDQ [20].

### Other properties

One [22] research reported the Responsiveness of the ASQOL questionnaire. Each one study evaluated the responsiveness of CASQ-QoL [30] questionnaire and the PGI [27]. Five articles [18, 20, 27, 30, 31] reported the floor and ceiling effects and missing data for ASQOL questionnaire, the PGI, and the CASQ-QoL questionnaire (Table 3).

**Table 3:** Meta-analysis of the ASQOL and EASi-QoL questionnaires

Outcomes	No. of studies	*Κ*	ES	[95% CI]
Internal consistency of the ASQOL questionnaire	11	20	0.48	[0.43, 0.52]
Internal consistency of the EASi-QoL questionnaire	2	8	0.46	[0.42, 0.49]
Test–retest reliability of the ASQOL questionnaire	4	12	1.26	[1.10, 1.41]
Construct validity of the ASQOL questionnaire (correlations with BASDAI)	3	3	1.05	[0.96, 1.16]
	4	5	0.64	[0.56, 0.73]
construct validity of the ASQOL questionnaire (correlations with BASFI)	9	18	0.74	[0.65, 0.84]
Analysis of construct validity of the EASi-QoL questionnaire	1	4	0.88	[0.78, 0.98]
	1	4	0.86	[0.76, 0.96]

ASQOL, the ankylosing spondylitis quality of life questionnaire; EASi-QoL, the evaluation of ankylosing spondylitis quality of life questionnaires

## Discussion

This is the first PRISMA-compliant systematic review and meta-analysis for measurement properties of QoL in AS/nr-SpA populations. In this systematic review, 11 identified QoL questionnaires in 22 publications were summarized and the reliability and validity of different questionnaires were outlined. Reliability could represent the consistency, stability of scales at various times and populations. Validity is an estimate of the validity and accuracy of the test, which including content validity, construct validity, and structural validity. This systematic review suggested that the identified questionnaires have generally excellent internal consistency, test–retest reliability, and usually had moderate or good convergent validity. The ASQOL questionnaire was the most widely studied questionnaire. This questionnaire was initially developed parallelly in the United Kingdom and the Netherland, on the basis of a conceptual model. The Cronbach’s α coefficient and ICC were highest for it. The next most commonly used tool was the EASi-QoL questionnaire. The ICC was highest for the physical function domains. The SF-36 survey contains 36 items divided into eight domains, covering physical, social function, and mental health. It is convenient for researchers to use in comparison of the QoL among individuals in different health conditions. However, only one included study [26] paid attention to the measurement properties in the Singapore population. Convergent validity and discriminative validity were also variable for the clinical measures. The assessment of convergent validity and discriminative validity demonstrated a strong correlation of QoL questionnaires with disease activity measures. Fatigue, pain or chest expansion, recognized symptoms of AS, also showed a moderate association of QoL. Besides, BASDAI and BASFI are generally considered as validity criteria. Our results showed that included scales and the constructs could better reflect the multifaceted features of disease activity and health-related QoL in AS/nr-SpA patients. Meanwhile, the ASQOL questionnaire was also frequently used as an accepted disease-specific QoL scales for evaluation of QoL in AS/nr-SpA patients. Other measurements properties such as responsiveness have also been reported in some publications. Furthermore, it should be noted that quality of evidence for included studies was low to high.

PROMs properties are recommended to be evaluated by the COSMIN checklist. The COSMIN criteria could be used as a guideline to help selecting the most appropriate health state measurement tools in research and clinical practice in systematic review. According to the COSMIN standards, most instruments had adequate methodological quality. The meta-analysis showed that the ASQOL and EASi-QoL questionnaires both had strong reliability and moderate validity. Many effect indicators would correlate to the methodological quality of PROMs properties. For example, if the sample size was huge (more than 200 patients when using IRT/Rasch analysis model), or the time interval was appropriate (usually two weeks), or patients enrolled were stable, there will be “very good” methodological quality. If one study only reported the ICC without clear descriptions, “adequate” will be evaluated as that in test–retest reliability. Some studies didn’t perform the classical test theory or the IRT/Rasch analyses model to assess the structural validity, thus “doubtful” or “inadequate” will be rated to the structural validity.

On the basis of current studies, high heterogeneity was displayed in different countries and languages, especially these non-native English countries. Subgroup analysis was frequently used to explore the heterogeneity of meta-analysis. Although the ASQOL questionnaire and the SF-36 survey had been validated and used in countries worldwide. It still hard to overcome cultural and linguistic differences between countries. With the diversity of expression habits and customs, it is vital that researchers should develop translation and adoption studies in various languages versions. Few articles investigated the translation and adoption of the ASQOL and EASi-QoL questionnaires in Asian by using the COSMIN standard. With the increasing attention to QoL of patients with AS/nr-SpA, more concentrations should be put on measurement properties of these disease-specific QoL instruments in Asian countries. The SQOL-AS scale was designed and adopted in Chinese population. However, the reliability and validity should be confirmed with a lager sample size. There was a limit of consistency in statistic analysis among different articles. ICC was calculated to represent the internal consistency in some researches while the others used the Spearman’s or Pearson’s correlation coefficients. This might be due to differences in methods and outcomes across studies, including, but not limited to the heterogeneity of disease activity and disease durations.

There was only one review [35] focused on factors associated with QoL has systematically summarized the instruments for evaluating the QoL of AS patients. Several meta-analyses have been designed to calculate the QoL scores or predict the disease-related factors.

Despite this, there remains some limitations. This systematic review didn’t focus on content validity because only a few studies reported details about this property. Only published studies could be included in this systemic review. Although we tried to scan all the potential studies about the QoL questionnaires in AS/nr-SpA patients, the incompleteness of information could not be ignored. There was no result of the grey literatures after electronic searching and checking the reference lists. In this systematic review, most studies had an unclear selection bias with the cross-sectional study design and some studies didn’t report the randomized strategy. The validity criteria varied in included articles. Only data of three criteria (the BASDAI, BASFI and ASQOL questionnaire) was pooled in this meta-analysis to represent the construct validity. The Fisher method was used to meet the need to determine variance in analysis when data was not directly reported. Importantly, the previous researches on documented the lack of sufficient comparisons of these instruments in the same population, this unmet need should also be filled by future qualitative research. For the same reason of quantities limitation or high heterogeneity, the meta-analysis was only performed in two scales. Thus, the conclusions still need to be confirmed by high-quality studies.

## Conclusion

This study indicated that the ASQOL and the EASi-QoL questionnaires are validated, reliable disease specific questionnaires for assessment of QoL in patients with AS/nr-axSpA. Different questionnaires have different clinical characteristics and measurement properties. Data from QoL studies are conflicting. Cultural and linguistic differences between countries should be considered during a new QoL questionnaire adoption. Future qualitative researches are also needed to compare different scales in measurement properties.

## Supplementary Information

**Additional file 1.** Search Terms for used English database.

**Additional file 2.** PROMs properties, GRADE and methodological quality evaluation, effect sizes of meta-analysis.

**Additional file 3.** Risk of bias graph and funnel plots.

## Data Availability

All data generated or analyzed during this study are included in this published article and its supplementary information files (Additional files 1 and 2).
