# Supplementary material for: The validity and reliability of quality of life questionnaires in patients with ankylosing spondylitis and non-radiographic axial spondyloarthritis: a systematic review and meta-analysis
Source: Health Qual Life Outcomes. 2022 Jul 30;20:116. doi: 10.1186/s12955-022-02026-5 (PMC9338652; doi:10.1186/s12955-022-02026-5)
Supplement: Supplementary file 1 — Additional file 1. Search Terms for used English database. [file 12955_2022_2026_MOESM1_ESM.doc]

Appendix 1 Search Terms

PUBMED

#1 MeSH Major Topic=Spondylitis, Ankylosing

#2 MeSH Major Topic=axial spondyloarthritis

#3 MeSH Major Topic=ankylosing spondylitis

#4 MeSH Major Topic=quality of life

#5 MeSH Major Topic=reliability

#6 MeSH Major Topic=validity

#7 MeSH Major Topic=internal consistency

#8 MeSH Major Topic=questionnaires

#9 MeSH Major Topic=surveys

#10 MeSH Major Topic=scales

#11 MeSH Major Topic=index

#12 MeSH Major Topic=SF-36

#13 MeSH Major Topic=short forms

#14 #1 OR #2 OR #3

#15 #5 OR #6 OR #7 OR #8 OR #9 OR #10 OR #11 OR #12 OR #13

#16 #14 AND #4 AND #15

Up to 2020-10-31

EMBASE

| #1.'spondylitis, ankylosing'/exp/mj OR 'axial spondyloarthritis'/exp/mj OR 'ankylosing spondylitis'/exp/mj | 22,433 |
| --- | --- |
| #2.'quality of life'/exp/mj | 113,101 |
| #3.'questionnaires'/exp/mj OR 'surveys'/exp/mj OR scales OR 'index'/exp/mj OR 'reliability'/exp/mj OR 'validity'/exp/mj OR 'internal consistency'/exp/mj OR forms OR 'sf36'/exp/mj OR 'short forms' | 823,294 |
| #4. questionnaires OR surveys OR scales OR index OR reliability OR validity OR (internal AND consistency) OR forms OR sf36 OR (short AND forms) | 3,536,830 |
| #5. #3 OR #4 | 3,550,096 |
| #6. #1 AND #2 AND #5 | 248 |

Cochrane Library

| #1 MeSH =Spondylitis, Ankylosing | 2196 |
| --- | --- |
| #2 MeSH =axial spondyloarthritis | 627 |
| #3 MeSH =ankylosing spondylitis | 2196 |
| #4 #1 OR #2 OR #3 | 2381 |
| #5 MeSH =quality of life | 126191 |
| #6 #4 AND #5 | 473 |
| #7 MeSH =questionnaires | 606560 |
| #8 MeSH =surveys | 34941 |
| #9 MeSH =scales | 41467 |
| #10 MeSH =index | 169378 |
| #11 MeSH =short forms | 4706 |
| #12 MeSH =SF-36 | 11846 |
| #13 MeSH =reliability | 12240 |
| #14 MeSH =validity | 14346 |
| #15 MeSH =internal consistency | 4825 |
| #16 #7 OR #8 OR #8 OR #9 OR #10 OR #11 OR #12 OR #13 OR #14 OR #15 | 270371 |
| #17 #16 AND #16 | 383 |
