# Supplementary material for: The validity and reliability of quality of life questionnaires in patients with ankylosing spondylitis and non-radiographic axial spondyloarthritis: a systematic review and meta-analysis
Source: Health Qual Life Outcomes. 2022 Jul 30;20:116. doi: 10.1186/s12955-022-02026-5 (PMC9338652; doi:10.1186/s12955-022-02026-5)
Supplement: Supplementary file 2 — Additional file 2. PROMs properties, GRADE and methodological quality evaluation, effect sizes of meta-analysis. [file 12955_2022_2026_MOESM2_ESM.doc]

Appendix 2


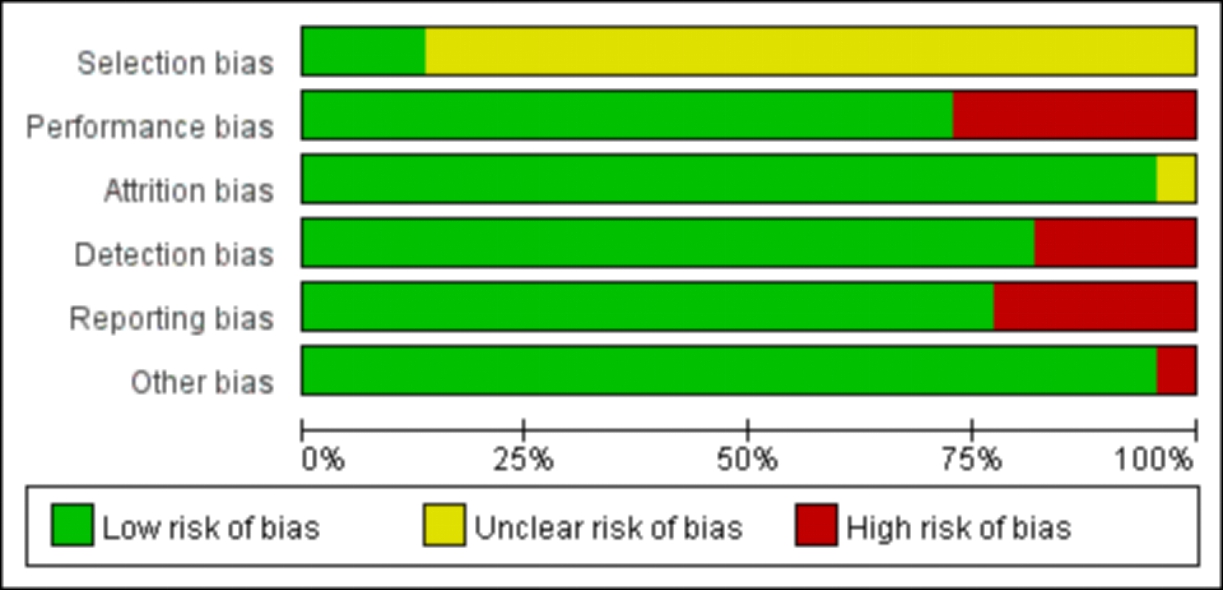


Figure1 Risk of bias graph


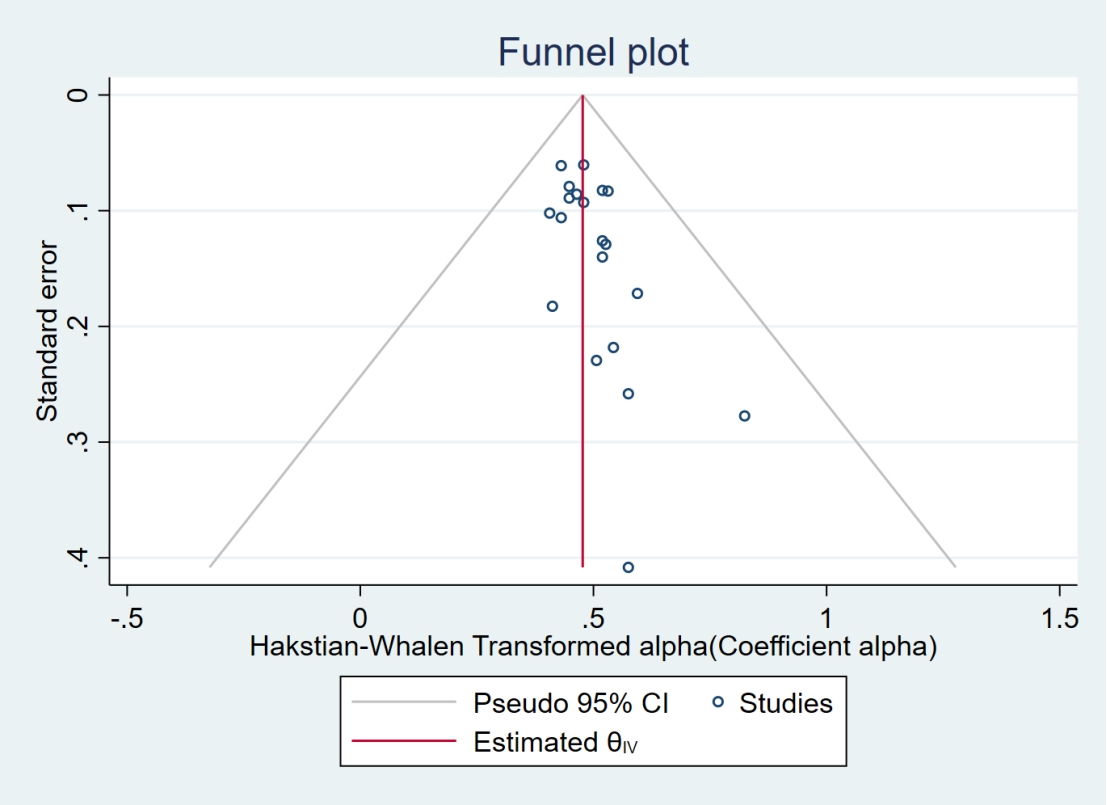


Figure 2 Funnel plots for the internal consistency of the ASQOL questionnaire


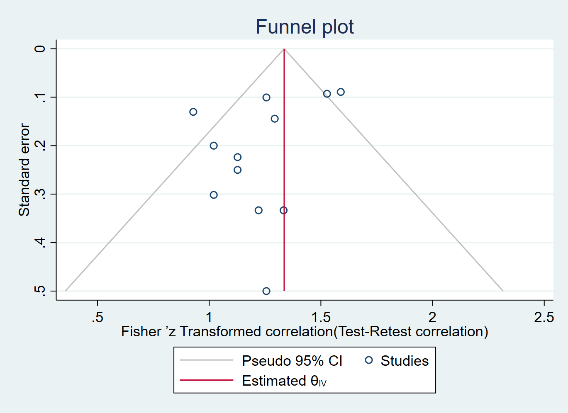


Figure 3 Funnel plots for test-retest reliability of the ASQOL questionnaire

Abbreviation: Left: Spearman’s correlation coefficient. Right: ICC, intraclass correlation coefficient.


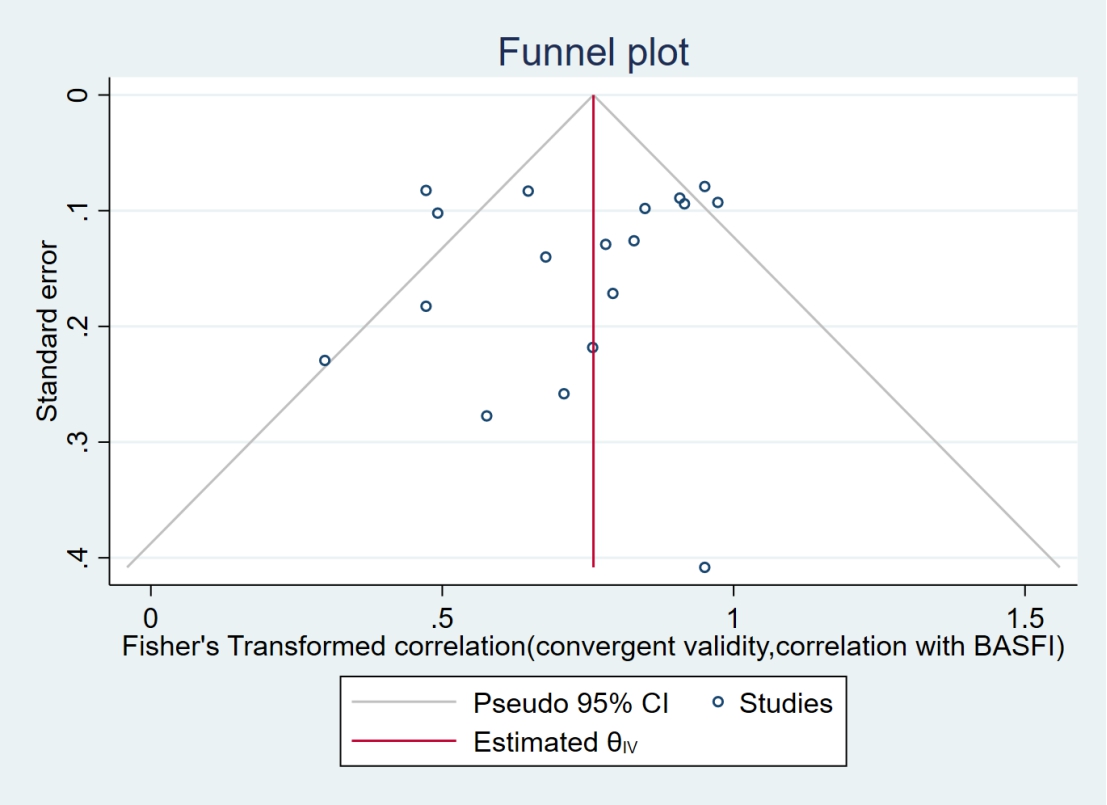


Figure 4 Funnel plots for correlations of the ASQOL questionnaire and BASFI.

Abbreviations: CI: confidence interval; BASFI: Bath Ankylosing Spondylitis Functional Index.

Table 1 Summery included PROMs properties

| **PROMs* (Ref)** | **No. of items (Recall period)** | **Reliability** | **Structrural** | **Other properties** |
| --- | --- | --- | --- | --- |
| ASQOL (8) | 18 (0-18) | Both language versions had excellent internal consistency (a=0.89–0.91), test-retest reliability (r_s_=0.92 UK and r_s_=0.91 NL), and validity. | Rasch analysis of data from the second survey (UK: n=164; NL: n=154) showed some item misfit, but showed that items formed a hierarchical order and were stable over time. Problematic items were removed giving an 18 items scale. Moderate to high correlations were found between the ASQOL and all the comparator instruments. | - |
| EuroQol (14) | 5; 12 (NR) | Test–retest reliability estimates support the use of both instruments in group evaluation and the  SF-12 Physical Component Summary score (PCS) in individual evaluation (>0.90). | Moderate correlations between both sections of the EuroQol and the SF-12 PCS were found. This was as hypothesized, as the majority of the EuroQol questions are concerned with physical health. Small to moderate correlations were found between both sections of the EuroQol and the SF-12 MCS. A very small correlation between the two sections of the SF-12, and a moderate to large correlation between the EQ-5D and EQ-VAS was found. | - |
| SF-12 (14) | 12 (NR) |
| ASQOL (15) | 18 (0-18) | Internal Consistency: Cronbach’s alpha =0.854，Test-retest reliability: rho=0.73 | the Spearman’s correlation coefficient with BASFI is 0.635, with BASDAI is 0.521, and with patient global VAS scores is 0.546 | It uses a needs-based quality of life model, which assumes that the quality of life is obtained from the individual's ability to meet their own needs. It can monitor patients from the perspective of patients and evaluate the therapeutic effects of new drugs. |
| ASQOL (16) | 18 (0-18) | Internal Consistency: Cronbach’s alpha =0.44-0.87，Test-retest reliability: r=0.77-0.96 | Logistic Item Response Theory (IRT) model: the correlation coefficient with BASFI is 0.57, and the correlation with SF-36 is moderate.  Classical psychometric theory evaluates convergence validity (correlation between ASQL and NHP, BASFI and BASDAI) and discriminative validity (correlation between ASQL and disease severity and overall health). The correlation with morning stiffness is the most significant. ASQL has a good correlation with finger base test and chest expansion, and has a moderate correlation with the number of painful areas and the number of affected areas. | The questionnaire covers physical function, social function, role-body, role-emotion, mental health, vitality, pain and overall health perception, etc. |
| ASQOL (17) | 18 (0-18) | Internal Consistency: Cronbach’s alpha =0.89，Test-retest reliability: ICC=0.96 | Classical psychometric theory evaluates convergence validity (correlation between ASQOL and NHP, BASFI and BASDAI) and discriminative validity (correlation between ASQOL and disease severity and overall health). The correlation with morning stiffness is the most significant. ASQOL has a good correlation with finger base test and chest expansion, and has a moderate correlation with the number of painful areas and the number of affected areas. | - |
| ASQOL (18) | 18 (0-18) | Internal Consistency: Cronbach’s alpha =0.93 (Chinese)；0.86 (English) ，Test-retest reliability: ICC=0.86 | Unidimensionality, Residual principal component analysis: 37.2% of the residual variance can be explained by the Rasch model. There is a moderate correlation with SF-36 scores in various fields and total scores | - |
| ASQOL (19) | 18 (0-18) | Internal Consistency: Cronbach’s alpha=0.86，Test-retest reliability of 12 items: Kappa=1，Rho=0.98 | The ASQOL scores had high correlations with physical (rho = 0.79) and mental (0.69) SF-36 components, the SF-36 domains pain (0.82), vitality (0.75), and role-physical (0.68), and the most of the disease-related variables. | - |
| ASQOL (20) | 18 (0-18) | Cronbach’s alpha ranged from 0.87 (BASDAI) to 0.93 (RLDQ). Test–retest reliability estimates support the use of the ASQOL in individual evaluation (>0.90) | PCA supported instrument unidimensionality. Correlations between instruments were in the hypothesized direction; the largest was between the ASQOL and BASDAI (0.79). The BASDAI had the strongest linear relationship, with responses  to both specific and general health transition questions (P<0.01). | The RLDQ had low levels of responsiveness. |
| RLDQ (20) | 16(NR) | Internal Consistency: Cronbach’s alpha=0.93，Test–retest reliability estimates support the use of the RLDQ in individual evaluation (>0.90). | Item responses for the RLDQ were skewed towards higher levels of functional ability. |
| ASQOL (21) | 18 (0-18) | Test–re-test reproducibility for ASQOL was good as assessed by intra-class correlation coefficient (ICC: 0.97, P < 0.001). Internal consistency was  high (Cronbach’s alpha: 0.91). | Convergent validity was confirmed by correlation of ASQOL score with specific scales (BASFI, r = 0.74, BASDAI, r = 0.6, fatigue, r = 0.56, depression, r = 0.24, intermalleolar distance, r = −0.44 and educational level, r = −0.37). | - |
| ASQOL (22) | 18 (0-18) | Internal Consistency: Cronbach‘s α=0.9，Test-retest reliability: ICC=0.89 | A 2-parameter Rasch model confirmed unidimensionality (chi-square fit p=0.86) with good item discrimination. Convergent validity was ascertained by high correlation of ASQOL score with disease activity measures (r=0.57 to 0.79). | Responsiveness was moderate (SRM 0.44) in patients improving and good (SRM 0.68) in patients worsening over the period. |
| ASQOL (23) | 18 (0-18) | NR | There were significantly correlations of changing between ASQOL and BAS-G, BASDAI and BASFI after treatment with etanercept in AS patients. | The Chinese ASQOL questionnaire is valuable to  evaluate the activity of AS patients and effect of biologic agent treatment in patients with AS. |
| ASQOL (24) | 18 (0-18) | Internal consistency was good (Cronbach: 0.933; IC95%=0.86-0.95). intra-class coefficient: 0.87 (IC 95%: 0.79-0.92). | ASQOL was significantly correlated with BASDAI (p<0.001), BASFI (p<0.001), BASG-s (p<0.001), BASMI (p=0.026), MASES (p=0.024) and all items of SF-36. | The mean time to complete the questionnaire was 5 minutes. |
| ASQOL (25) | 18 (0-18) | The ASQOL total score had a high internal consistency (ωH = 0.82) within this population. Test–retest reliability: ICC estimates that exceeded the prespecified criterion  of 0.7. | Concurrent validity of the ASQOL at Baseline exceeded  the prespecified criterion for acceptable validity for all validators  (r ≥ |0.50|), excepting PhGADA and ASDAS composite  score (r = 0.24 and r = 0.34, respectively). |  |
| SF-36(26) | 36 (0-100) | SF-36 scales showed high internal consistency ranging from 0.88 to 0.90. | Construct validity was supported as shown by fulfillment  of all hypotheses. Divergent construct validity was supported, as SF-36 MCS was not associated with PGA, pain and HAQ. | - |
| Patient Generated Index，PGI (27) | NR | Test-retest reliability estimates support the use of the PGI in group evaluation (ICC > 0.80). | It has the greatest correlation with ASQOL, the least correlation with disease-specific tools, and the least correlation with RLDQ. |  |
| EASI-QOL (28) | 20 items, 4 dimensions (0-16) | Internal consistency: Cronbach’s alpha coefficient was 0.910 in the physical function category, 0.893 in the disease activity category, 0.935 in the emotional wellbeing category, and 0.930 in the social contribution category (α>0.7). Intra-class correlation coefficient value that was used for the reliability analysis of the Turkish EASi-QoL was 0.993. | Factor loadings of all items in EASi-QoL were higher than 0.7. There was also a strong positive correlation between all subcategories of EASi-QoL (physical function, disease  activity, emotional well-being and social participation) and AS-specific QoL scale, BASDAI, Bath BASFI, patient’s global assessment and pain severity. MASES and BASMI showed a moderate correlation with emotional well-being and social participation categories of EASi-QoL. The results of the explanatory factor analysis indicated that the factor loadings were higher than 0.40 for all items. CFI and TLI values higher  than 0.95 indicated a good fit and RMSEA values close to 0.08 indicated an acceptable fit. CFA shows the factor loadings of physical function, disease activity, emotional well-being, and social participation dimensions of EASi-QoL, which were acceptable for all items being higher than 0.7. | These are physical function (1-6 items), disease activity (7-10 items), emotional well-being (11-15 items), and social participation (16-20 items). Each item is scored on a 5-point scale (0-4). The scores of four dimensions are calculated independently. Lower scores indicate better QoL |
| EASI-QOL (29) | 20 items, 4 dimensions (0-16) | Cronbach’s alpha and test-retest reliability estimates were 0.88–0.92 and 0.88–0.93, | Confirmatory factor analysis and as hypothesized, EASi-QoL domains had high correlations with AS-specific questionnaires measuring related constructs. The item-total score correlation coefficient is from 0.66 to 0.84. | - |
| CASQ-QoL (30) | 10 (0-3) | The CASQ was also reliable (Cronbach’s alpha for CASQ-FI 0.958, and CASQ-QoL 0.966) and had no misfitting items. ICC for agreement demonstrated a quite good reliability of both CASQ-FI and CASQ-QoL (ICC were 0.96 and 0.96 respectively). | The CASQ questionnaire for functional impairment and quality of life showed acceptable validity as it correlated significantly with clinical parameters of disease activity: BASFI (CASQ-FI: r=0.85, CASQ-QoL: r=0.86), BAS-DAI (CASQ-FI: r=0.71, CASQ-QoL: r=0.87) and BAS-G (CASQ-FI: r=0.64, CASQ-QoL: r=0.79). | Both CASQ questionnaires were sensitive to change (p<0.01) |
| ASQOL (31) | 18 (0-18) | The measure had good internal consistency (α =0.92) and test-retest reliability (r =0.98). | Predicted correlations with the NHP provided evidence of the convergent validity of the two measures. Construct validity was confirmed by the measure’s ability to distinguish groups of AS patients varying by perceived disease severity and general health. | - |
| SQOL-AS (32) | 37(NR) | Internal consistency (Cronbach’s α＞0.75) , split-half reliability: item-total coefficient (r=0.933) ，test-retest reliability: the correlation coefficient ＞0.732 | The correlation of each item and its dimension scores is large (the correlation coefficient is greater than 0.6). | - |
| EQ-5D、SF-6D(33) | 5 dimensions、6 dimensions(NR) | Agreement (ICC) was moderate (0.46–0.55). | Correlation with external standards health status was similar  and was moderate to good for all external standards, with the  best correlations with ASQOL | - |
| Modified AS-AIMS2(34) | 13(NR) | high internal consistency (Cronbach’s α=0.78–0.91), and good reproducibility in the 13 dimensions and 5 components (ICC: 0.7–0.9). | Principal component analyses on AS-AIMS2 showed  satisfactory dimensionality (74.1% of variance explained), significant convergent validity of the new dimension with spine pain and mobility. Convergent validity of dimensions (Pearson correlation coefficients) was documented by a  fair to moderate correlation of increasing Schober  index scores with decreasing scores in self-care (r 5  0.31), arm movements (r=0.38), and spine mobility  (r=0.24). | The AIMS2 is a self-report health status questionnaire comprising 57 core items organized in 12 health dimensions, further  aggregated into 5 components by averaging the scores of the corresponding dimensions, namely Physical, Affect, Symptoms, Role, and Social Interaction. |

Abbreviation: ASQOL: the ankylosing spondylitis quality of life questionnaire; EASi-QoL: the evaluation of ankylosing spondylitis quality of life questionnaire; RLDQ: the revised Leeds disability questionnaire; CASQ-QOL: the combined AS questionnaire for quality of life questionnaire; PGI: the patient-generated index; SF-36: the short form-36 health survey; SF-12: the short form-12 health survey; modified AS-AIMS2: the ankylosing spondylitis-arthritis impact measurement scales 2; SQOL-AS: an ankylosing spondylitis patient quality of life measurement scale. PF: physician function; DA: disease activity; EW: emotional well-being; SP: social participation; ICC: intraclass correlation coefficient; RCTs: randomized controlled trials; BASDAI: Bath Ankylosing Spondylitis Disease Activity Index; BASFI: Bath Ankylosing Spondylitis Functional Index.

Table 2 Summary of Findings

| **Quality assessment for properties of ASQOL questionnaire** | | | | | | | | | | | | **Summary of Findings** | |
| --- | --- | --- | --- | --- | --- | --- | --- | --- | --- | --- | --- | --- | --- |
| **Participants (studies) Follow up** | **Risk of bias** | **Inconsistency** | | **Indirectness** | | **Imprecision** | | **Publication bias** | | **Overall quality of evidence** | | **Relative effect** (95% CI) | |
| **Internal consistency** (measured with: Cronbach’s alpha; Better indicated by lower values) | | | | | | | | | | | | | |
| 1929 (12 studies) | no serious risk of bias^1^ | no serious inconsistency | | no serious indirectness | | no serious imprecision | | undetected | | ⊕⊕⊕⊕ **HIGH**^1,3^ due to large effect | | 0.89  (0.86 to 0.92) | |
| **Reliability** (measured with: ICC/r; Better indicated by lower values) | | | | | | | | | | | | | |
| 590 (4 studies) | no serious risk of bias^1^ | no serious inconsistency | | no serious indirectness | | no serious imprecision | | undetected | | ⊕⊕⊕⊕ **HIGH**^1,3^ due to large effect | | 0.85  (0.80 to 0.89) | |
| **Validity** (Better indicated by lower values) | | | | | | | | | | | | | |
| 1650 (10 studies) | Serious^2^ | Serious^4^ | | no serious indirectness | | no serious imprecision | | undetected | | ⊕⊕⊕⊝ **MODERATE**^2,3,4^ due to risk of bias, inconsistency, large effect, plausible counfounding would change the effect | | BASDAI:0.78  (0.74 to 0.82])  0.56  (0.50 to 0.62)  0.31  ([0.14, 0.46])  BASFI:0.62  (0.57 to 0.68) | |
| **Quality assessment for properties of EASi-QOL questionnaire** | | | | | | | | | | | | | **Summary of Findings** |
| **Participants (studies) Follow up** | **Risk of bias** | | **Inconsistency** | | **Indirectness** | | **Imprecision** | | **Publication bias** | | **Overall quality of evidence** | | **Relative effect** (95% CI) |
| **Internal consistency** (measured with: Cronbach’s alpha; Better indicated by lower values) | | | | | | | | | | | | | |
| 712 (2 studies) | no serious risk of bias^1^ | | no serious inconsistency | | no serious indirectness | | no serious imprecision | | undetected | | ⊕⊕⊕⊕ **MODERATE** ^1,3^ due to large effect | | 0.91  (0.88 to 0.93) |
| **Validity** (Better indicated by lower values) | | | | | | | | | | | | | |
| 712 (2 studies) | Serious^2^ | | no serious inconsistency | | no serious indirectness | | no serious imprecision | | undetected | | ⊕⊕⊕⊝ **MODERATE**^2,3^ due to risk of bias, inconsistency, large effect, plausible counfounding would change the effect | | 0.75～0.86  (0.68 to 0.88) |
| **CI:** Confidence interval; **MD:** Mean difference; **OR:** Odds ratio; **ASQOL**: the ankylosing spondylitis quality of life questionnaire; **EASi-QoL**: the evaluation of ankylosing spondylitis quality of life questionnaire; **BASDAI**: Bath Ankylosing Spondylitis Disease Activity Index; **BASF**I: Bath Ankylosing Spondylitis Functional Index.  **Explanation:**  1 No-There are multiple studies of at least adequate quality, or there is one study of very good quality available  2 Serious-There are multiple studies of doubtful quality available, or there is only one study of adequate quality 3 Large effect size (RR/OR＞5 or RR＜0.2) and no apparent publication bias or imprecision. 4 There are inconsistent results for structural validity which cannot be explained. | | | | | | | | | | | | | |

Table 3 Methodological quality evaluation.

| **Ref** | **Structural validity** | | | **Internal consistency** | | | **Reliability** | | | | **Criterion validity** | | | **Hypotheses testing for construct validity** | | | **Comparison between subgroups (discriminative or known-groups validity)** | | | **Responsiveness** | | |
| --- | --- | --- | --- | --- | --- | --- | --- | --- | --- | --- | --- | --- | --- | --- | --- | --- | --- | --- | --- | --- | --- | --- |
|  | 1 | 2 | total | 1 | 2 | total | 1 | 2 | total | | 1 | 2 | total | 1 | 2 | total | 1 | 2 | total | 1 | 2 | total |
| [8] | A | A | A | V | V | V | A | A | | A | V | V | V | V | V | V | A | A | A | - | - | - |
| [14] | A | A | A | - | - | - | A | A | | A | - | - | - | - | - | - | - | - | - | - | - | - |
| [15] | A | D | D | V | V | V | V | V | | V | - | - | - | V | V | V | - | - | - | - | - | - |
| [16] | A | A | A | V | V | V | V | V | | V | V | V | V | V | V | V | - | - | - | - | - | - |
| [17] | V | V | V | V | V | V | A | A | | A | - | - | - | - | - | - | - | - | - | - | - | - |
| [18] | A | I | A | V | A | V | V | A | | V | V | V | V | - | - | - | - | - | - | - | - | - |
| [19] | I | I | I | V | V | V | A | D | | D | I | I | I | V | V | V | - | - | - | - | - | - |
| [20] (ASQOL) | A | A | A | V | V | V | V | V | | V | - | - | - | V | V | V | - | - | - | - | - | - |
| [20] (RLDQ) | A | A | A | V | V | V | V | V | | V | - | - | - | V | V | V | - | - | - | - | - | - |
| [21] | I | I | I | V | V | V | A | A | | A | V | A | V | V | A | A | - | - | - | - | - | - |
| [22] | A | A | A | V | V | V | D | A | | D | - | - | - | - | - | - | V | V | V | - | - | - |
| [23] | I | I | I | - | - | - | - | - | | - | - | - | - | - | - | - | - | - | - | - | - | - |
| [24] | I | I | I | V | V | V | V | V | | V | - | - | - | - | - | - | - | - | - | - | - | - |
| [25] | V | V | V | V | V | V | V | V | | V | - | - | - | - | - | - | - | - | - | - | - | - |
| [26] | V | V | V | V | V | V | V | V | | V | - | - | - | - | - | - | - | - | - | - | - | - |
| [27] | I | I | I | - | - | - | V | V | | V | - | - | - | - | - | - | - | - | - | - | - | - |
| [28] | A | A | A | V | V | V | V | V | | V | V | V | V | V | V | V | - | - | - | - | - | - |
| [29] | A | A | A | V | V | V | A | A | | A | - | - | - | V | V | V | - | - | - | - | - | - |
| [30] | A | A | A | V | V | V | V | V | | V | V | V | V | - | - | - | - | - | - | - | - | - |
| [31] | A | A | A | V | V | V | V | V | | V | - | - | - | - | - | - | - | - | - | - | - | - |
| [32] | I | I | I | V | V | V | - | - | | - | - | - | - | - | - | - | - | - | - | V | V | V |
| [33] | I | I | I | D | D | D | V | V | | V | V | V | V | - | - | - | - | - | - | - | - | - |
| [34] | D | D | D | V | V | V | D | V | | D | I | I | I | - | - | - | - | - | - | - | - | - |

Abbreviation: V=Very good, A=Adequate, D=Doubtful, I=Inadequate, “-” = Not applicable.

Table 4 Meta-analysis of the ASQOL and EASi-QoL questionnaires.

| **Study** | **ES** | **[95% Conf.** | **Interval]** | **% Weight** |
| --- | --- | --- | --- | --- |
| **1. Analysis of the internal consistency of the ASQOL questionnaire** | | | | |
| **Jenks et al 2010 (New Zealand)** | 0.53 | 0.27 | 0.78 | 3.21 |
| **Doward et al 2007 (US)** | 0.53 | 0.37 | 0.69 | 7.76 |
| **Doward et al 2007 (Canadian English)** | 0.52 | 0.27 | 0.77 | 3.37 |
| **Doward et al 2007 (Canadian French)** | 0.82 | 0.28 | 1.37 | 0.70 |
| **Doward et al 2007 (French)** | 0.58 | 0.26 | 1.08 | 0.80 |
| **Doward et al 2007 (German)** | 0.59 | 0.06 | 0.93 | 1.82 |
| **Doward et al 2007 (Italian)** | 0.51 | 0.12 | 0.96 | 1.02 |
| **Doward et al 2007 (Spanish)** | 0.54 | -0.23 | 0.97 | 1.12 |
| **Doward et al 2007 (Swedish)** | 0.48 | 0.36 | 1.38 | 0.32 |
| **Duruöz et al 2012** | 0.58 | 0.07 | 0.69 | 14.66 |
| **Leung et al 2017 (Chinese)** | 0.41 | 0.05 | 0.77 | 1.61 |
| **Leung et al 2017 (Singapore English)** | 0.52 | 0.36 | 0.68 | 7.87 |
| **Ariza-Ariza et al 2006 (Spainish)** | 0.52 | 0.25 | 0.79 | 2.73 |
| **Pham et al 2010 (French)** | 0.46 | 0.30 | 0.63 | 7.28 |
| **Haywood et al 2002(2) (England)** | 0.43 | 0.31 | 0.55 | 14.34 |
| **Doward et al 2003 (the UK)** | 0.45 | 0.27 | 0.62 | 6.74 |
| **Doward et al 2003 (Netherlands)** | 0.48 | 0.30 | 0.66 | 6.21 |
| **Fallahi et al 2013 (Persian)** | 0.45 | 0.29 | 0.60 | 8.56 |
| **Graham et al 2015 (Greek)** | 0.43 | 0.22 | 0.64 | 4.76 |
| **Hamdi et al 2012 (Tunisian)** | 0.41 | 0.21 | 0.61 | 5.14 |
| **Overall** | 0.48 | 0.43 | 0.52 |  |
| 1. **Analysis of the internal consistency of the EASi-QoL questionnaire** | | | | |
| **Öncülokur et al 2018 (PF)** | 0.45 | 0.25 | 0.65 | 3.43 |
| **Öncülokur et al 2018 (DA)** | 0.47 | 0.28 | 0.67 | 3.43 |
| **Öncülokur et al 2018 (EW)** | 0.40 | 0.20 | 0.60 | 3.43 |
| **Öncülokur et al 2018 (SP)** | 0.41 | 0.21 | 0.61 | 3.43 |
| **Haywood et al 2010 (PF)** | 0.46 | 0.38 | 0.54 | 21.57 |
| **Haywood et al 2010 (DA)** | 0.49 | 0.41 | 0.57 | 21.57 |
| **Haywood et al 2010 (EW)** | 0.45 | 0.37 | 0.53 | 21.57 |
| **Haywood et al 2010 (SP)** | 0.43 | 0.35 | 0.51 | 21.57 |
| **Overall** | 0.46 | 0.42 | 0.49 |  |
| 1. **Analysis of theTest-retest reliability of the ASQOL questionnaire** | | | | |
| **Jenks et al 2010 (New Zealand)** | 0.93 | 0.47 | 0.99 | 8.60 |
| **Doward et al 2007 (US)** | 1.26 | 0.65 | 1.05 | 14.43 |
| **Doward et al 2007 (Canadian English)** | 1.29 | 0.58 | 1.14 | 7.00 |
| **Doward et al 2007 (Canadian French)** | 1.33 | 0.22 | 1.52 | 1.31 |
| **Doward et al 2007 (German)** | 1.02 | 0.38 | 1.16 | 3.64 |
| **Doward et al 2007 (Italian)** | 1.22 | 0.19 | 1.49 | 1.31 |
| **Doward et al 2007 (Spanish)** | 1.02 | 0.18 | 1.36 | 1.60 |
| **Doward et al 2007 (Swedish)** | 1.26 | -0.13 | 1.83 | 0.58 |
| **Leung et al 2017 (Chinese)** | 1.13 | 0.32 | 1.30 | 2.33 |
| **Leung et al 2017 (Singapore English)** | 1.13 | 0.37 | 1.25 | 2.92 |
| **Doward et al 2003 (the UK)** | 1.59 | 0.75 | 1.09 | 18.37 |
| **Doward et al 2003 (Netherlands)** | 1.53 | 0.73 | 1.09 | 16.91 |
| **Overall** | 1.26 | 1.10 | 1.41 |  |
| 1. **Analysis of construct validity of the ASQOL questionnaire (correlations with BASDAI)** | | | | |
| **(i)** **Ariza-Ariza et al 2006 (Spainish)** | 1.13 | 0.85 | 1.40 | 12.06 |
| **Pham et al 2010 (French)** | 1.07 | 0.88 | 1.26 | 24.59 |
| **Haywood et al 2002(1) (England)** | 1.03 | 0.91 | 1.15 | 63.36 |
| **Overall** | 1.05 | 0.96 | 1.16 |  |
| **(ii)** **Jenks et al 2010 (New Zealand)** | 0.58 | 0.32 | 0.83 | 11.76 |
| **Leung et al 2017 (Chinese)** | 0.55 | 0.19 | 0.91 | 5.88 |
| **Leung et al 2017 (Singapore English)** | 0.55 | 0.39 | 0.71 | 28.82 |
| **Fallahi et al 2013 (Persian)** | 0.69 | 0.54 | 0.85 | 31.37 |
| **Zhao et al 2007 (Chinese)** | 0.76 | 0.58 | 0.95 | 21.61 |
| **Overall** | 0.64 | 0.56 | 0.73 |  |
| 1. **Analysis of construct validity of the ASQOL questionnaire (correlations with BASFI)** | | | | |
| **Jenks et al 2010 (New Zealand)** | 0.78 | 0.53 | 1.03 | 5.63 |
| **Doward et al 2007 (US)** | 0.65 | 0.48 | 0.81 | 6.37 |
| **Doward et al 2007 (Canadian English)** | 0.83 | 0.58 | 1.08 | 5.68 |
| **Doward et al 2007 (Canadian French)** | 0.58 | 0.03 | 1.12 | 3.28 |
| **Doward et al 2007 (French)** | 0.71 | 0.20 | 1.21 | 3.53 |
| **Doward et al 2007 (German)** | 0.79 | 0.46 | 1.13 | 4.89 |
| **Doward et al 2007 (Italian)** | 0.30 | -0.15 | 0.75 | 3.94 |
| **Doward et al 2007 (Spanish)** | 0.76 | 0.33 | 1.19 | 4.12 |
| **Doward et al 2007 (Swedish)** | 0.95 | 0.15 | 1.75 | 2.02 |
| **Leung et al 2017 (Chinese)** | 0.47 | 0.11 | 0.83 | 4.70 |
| **Leung et al 2017 (Singapore English)** | 0.47 | 0.31 | 0.63 | 6.38 |
| **Ariza-Ariza et al 2006 (Spainish)** | 0.68 | 0.40 | 0.95 | 5.44 |
| **Pham et al 2010 (French)** | 0.85 | 0.66 | 1.04 | 6.14 |
| **Doward et al 2003 (the UK)** | 0.91 | 0.73 | 1.08 | 6.28 |
| **Doward et al 2003 (Netherlands)** | 0.97 | 0.79 | 1.15 | 6.22 |
| **Fallahi et al 2013 (Persian)** | 0.95 | 0.80 | 1.11 | 6.42 |
| **Zhao et al 2007 (Chinese)** | 0.92 | 0.73 | 1.10 | 6.21 |
| **Hamdi et al 2012 (Tunisian)** | 0.49 | 0.29 | 0.69 | 6.08 |
| **Overall** | 0.74 | 0.65 | 0.84 |  |
| 1. **Analysis of construct validity of the EASi-QoL questionnaire (correlations with BASDAI and BASFI)** | | | | |
| **(i)** **Öncülokur et al 2018 (PF)** | 0.86 | 0.66 | 1.06 | 25.00 |
| **Öncülokur et al 2018 (DA)** | 0.94 | 0.75 | 1.14 | 25.00 |
| **Öncülokur et al 2018 (EW)** | 0.87 | 0.67 | 1.07 | 25.00 |
| **Öncülokur et al 2018 (SP)** | 0.85 | 0.65 | 1.05 | 25.00 |
| **Overall** | 0.88 | 0.78 | 0.98 |  |
| **(ii) Öncülokur et al 2018 (PF)** | 0.97 | 0.77 | 1.17 | 25.00 |
| **Öncülokur et al 2018 (DA)** | 0.78 | 0.58 | 0.98 | 25.00 |
| **Öncülokur et al 2018 (EW)** | 0.78 | 0.58 | 0.98 | 25.00 |
| **Öncülokur et al 2018 (SP)** | 0.92 | 0.72 | 1.11 | 25.00 |
| **Overall** | 0.86 | 0.76 | 0.96 |  |

Abbreviation: ASQOL: the ankylosing spondylitis quality of life questionnaire; EASi-QoL: the evaluation of ankylosing spondylitis quality of life questionnaire; BASDAI: Bath Ankylosing Spondylitis Disease Activity Index; BASFI: Bath Ankylosing Spondylitis Functional Index; PF: physician function; DA: disease activity; EW: emotional well-being; SP: social participation.
