# Supplementary material for: The validity and reliability of quality of life questionnaires in patients with ankylosing spondylitis and non-radiographic axial spondyloarthritis: a systematic review and meta-analysis
Source: Health Qual Life Outcomes. 2022 Jul 30;20:116. doi: 10.1186/s12955-022-02026-5 (PMC9338652; doi:10.1186/s12955-022-02026-5)
Supplement: Supplementary file 3 — Additional file 3. Risk of bias graph and funnel plots. [file 12955_2022_2026_MOESM3_ESM.doc]

Appendix3 Risk of bias graph and funnel plots


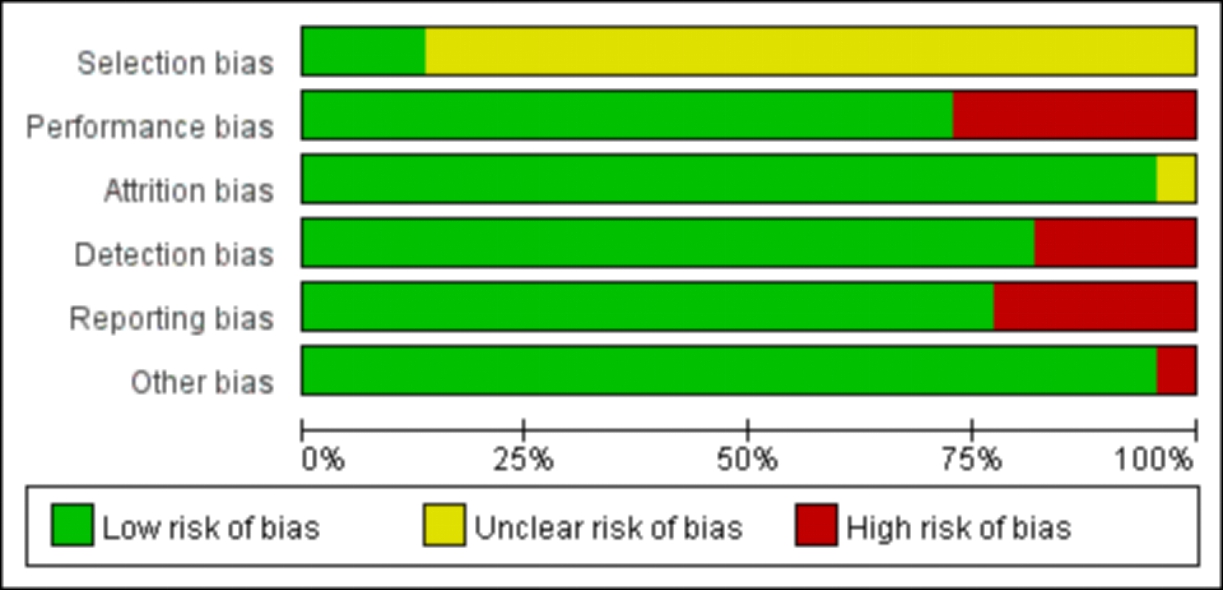


Figure1 Risk of bias graph


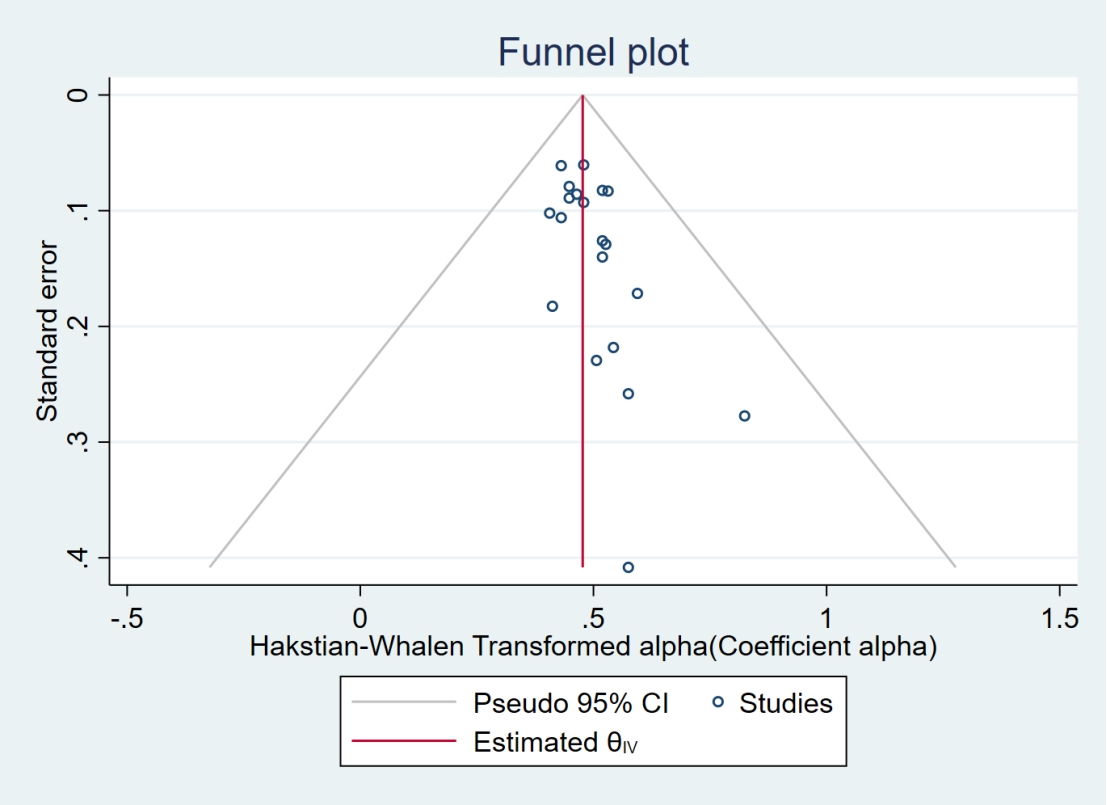


Figure 2 Funnel plots for the internal consistency of the ASQOL questionnaire


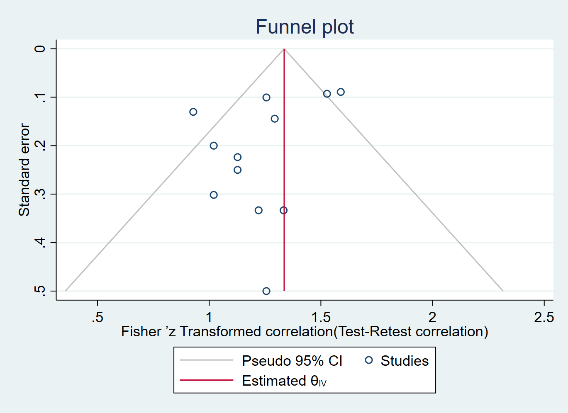


Figure 3 Funnel plots for test-retest reliability of the ASQOL questionnaire

Abbreviation: Left: Spearman’s correlation coefficient. Right: ICC, intraclass correlation coefficient.


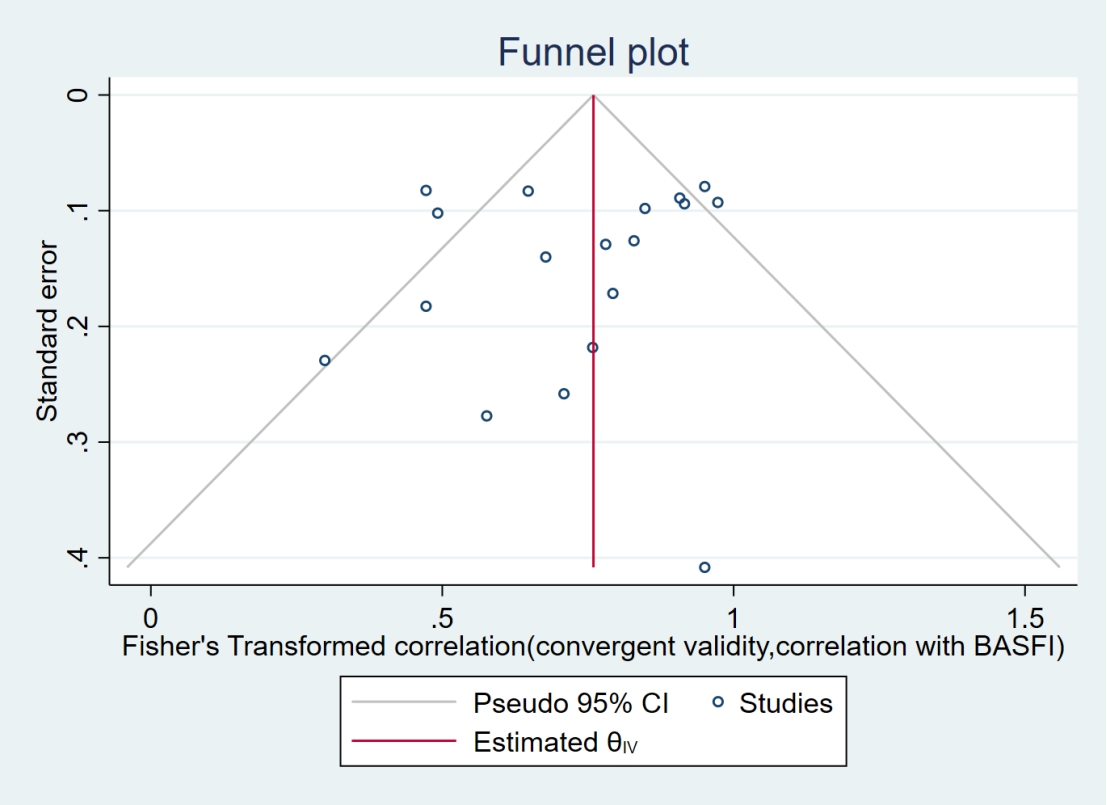


Figure 4 Funnel plots for correlations of the ASQOL questionnaire and BASFI.

Abbreviations: CI: confidence interval; BASFI: Bath Ankylosing Spondylitis Functional Index.
